# Comparative analysis among therapeutic modalities in ruptured hepatocellular carcinoma and identification of imaging predictors for survival

**DOI:** 10.1186/s12885-024-12829-y

**Published:** 2024-08-26

**Authors:** Natthaphong Nimitrungtawee, Phichayut Phinyo, Piemkamol Chalidapong, Nakarin Inmutto

**Affiliations:** 1 Department of Radiology, Diagnostic Radiology Unit, Faculty of Medicine, Chiang Mai University, Chiang Mai, Thailand; 2 Department of Family Medicine, Faculty of Medicine, Chiang Mai University, Chiang Mai, 50200 Thailand; 3 Center for Clinical Epidemiology and Clinical Statistics, Faculty of Medicine, Chiang Mai University, Chiang Mai, 50200 Thailand; 4 Musculoskeletal Science and Translational Research (MSTR), Chiang Mai University, Chiang Mai, 50200 Thailand

**Keywords:** Rupture hepatocellular carcinoma, Transarterial embolization, Perihepatic packing, Hepatectomy

## Abstract

**Background:**

Spontaneous rupture of hepatocellular carcinoma (rHCC) poses a life-threatening complication with a mortality rate of 25–75%. Treatment aims at achieving hemostasis and includes options such as trans-arterial embolization, perihepatic packing, and hepatic resection. The optimal treatment remains a subject of debate. Our retrospective review evaluates these treatments and investigates imaging’s role in prognosis for rHCC patients.

**Purpose:**

We aimed to compare survival outcomes among rHCC patients who received transarterial embolization (TAE), surgery (perihepatic packing, hepatectomy), or best supportive care (BSC), while also identifying predictive imaging factors in these patients.

**Materials and methods:**

All patients diagnosed with rHCC and admitted to Maharaj Nakorn Chiangmai Hospital between January 2012 and December 2021 were included. We reviewed clinical features, imaging results, treatment modalities, and outcomes. In order to balance pretreatment confounders, inverse probability treatment weighting (IPTW) was employed. Flexible parametric survival regression was utilized to compare survival outcomes and identify imaging factors predicting the survival of rHCC patients. Hazard ratios (HR) and the difference in restricted mean survival time (RMST) were reported.

**Result:**

Among the 186 rHCC patients included, we observed 90-day and 1-year mortality rates of 64% and 84%, respectively. Both the TAE and surgery groups exhibited significantly lower 1-year mortality rates compared to BSC. The HR were 0.56 (95% CI 0.33–0.96) for TAE and 0.52 (95% CI 0.28–0.95) for surgery compared to BSC. Both the TAE and surgery also significantly extended the 1-yeaar life expectancy post-initial treatment when compared to BSC, with an RMST difference of + 55.40 days (95% CI 30.18–80.63) for TAE vs. BSC and + 68.43 days (95% CI 38.77–98.09) for surgery vs. BSC. The presence of active contrast extravasation and bleeding in both lobes were independent prognostic factors for 1-year survival.

**Conclusions:**

TAE and surgical treatments provide comparable survival benefits for rHCC patients, extending survival time by approximately 2 months compared to best supportive care. We strongly recommend active management for all rHCC patients whenever possible.

**Supplementary Information:**

The online version contains supplementary material available at 10.1186/s12885-024-12829-y.

## Introduction

Hepatocellular carcinoma (HCC) is the most common primary liver cancer with a high mortality rate [1]. Ruptured hepatocellular carcinoma (rHCC) is a critical complication of HCC, exhibiting a higher prevalence in Asian and African countries compared with Western nations (3%-26% vs. 3%) [2, 3]. Due to the hypervascular nature of HCC [4], it can lead to tumor bleeding into the peritoneum, making rHCC a potentially life-threatening condition with a mortality rate ranging from 31 to 67% [5]. The median survival time of untreated rHCC is only about 1.2–4 months [6]. Despite advancements in medical technology, diagnosing and treating rHCC remain significant challenges [7, 8].

The primary goal in treating rHCC is to restore blood volume and stop bleeding, achievable through fluid resuscitation, blood transfusion, and coagulopathy correction [9]. Surgical methods, such as emergency surgical hemostasis (perihepatic packing) or transarterial embolization (TAE), are the current options for bleeding control in rHCC [10, 11]. However, the most effective treatment for rHCC remains controversial [12]. The primary aim of this study was to assess survival outcomes among the three available treatment options for rHCC patients: transarterial embolization, surgery, and best supportive care. Our secondary objective was to investigate imaging factors influencing survival time in patients with rHCC.

## Materials and methods

### Patient eligibility

This study has been granted approval by the Ethics Committee of the Faculty of Medicine, Chiang Mai University [RAD-2565–09175]. The need for informed consent was waived owing to the retrospective nature of data collection.

The electronic medical database was searched from January 2012 to December 2021. The inclusion criteria were patients who were at least 18 years old and diagnosed with rHCC. The diagnosis of rHCC was determined by a combination of clinical symptoms (abdominal pain, hypotension, bloody ascites from paracentesis) and a CT scan showing HCC with hemoperitoneum. The exclusion criteria were patients who received combined treatment (surgery with TAE) and patients with unavailable CT/MRI image data.

### Data collection

#### Baseline clinical information

Clinical data of all patients were retrospectively reviewed. These data included physical examination, hemorrhagic shock status class II or higher according to the advanced trauma life support (ATLS) guideline. [13], laboratory investigations (complete blood count, liver function tests, coagulogram), liver function assessment (Child-Turcotte-Pugh score), imaging studies (US, CT, MRI, angiogram), as well as the date of diagnosis and survival time.

### Imaging analysis

**Fig. 1:**
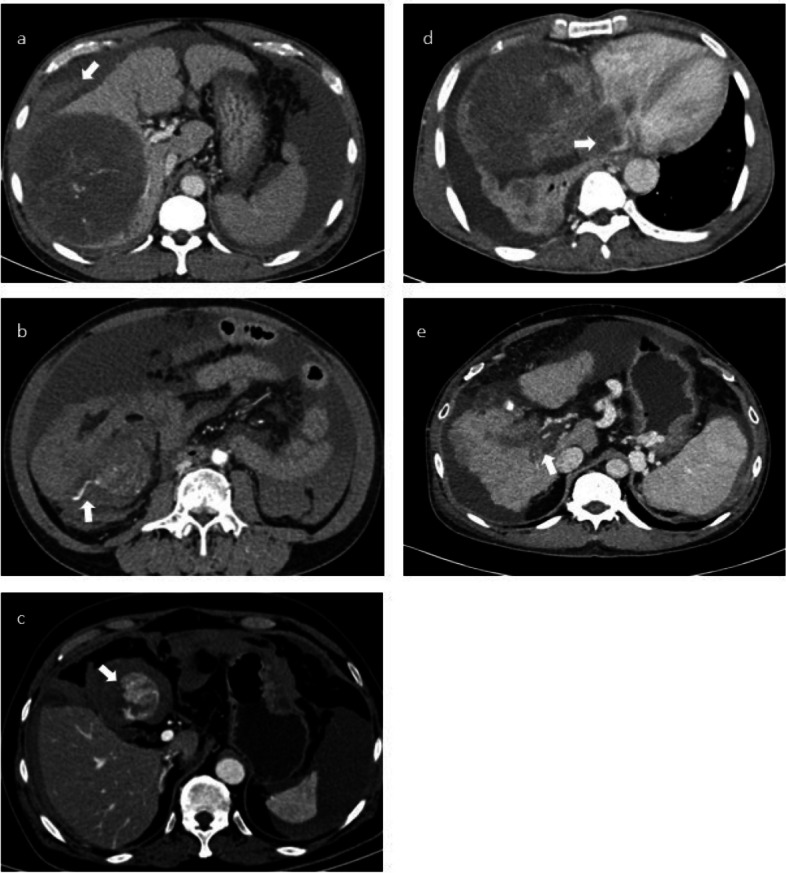
Image findings of rupture HCC. Sentinel clot sign (**a**), active contrast extravasation (**b**), tumor wall disruption (**c**), IVC invasion (**d**), Portal vein invasion (**e**)

The CT, MRI, and angiogram images were reviewed on the PACS using the Synapse workstation. Two interventional radiologists, one with 2 years of experience and the other with 10 years of experience, along with a third-year radiology resident, conducted this review based on consensus. Tumor size, the location of ruptured HCC, and non-ruptured HCC, along with other frequently observed ancillary imaging findings in rHCC such as active contrast extravasation, sentinel clot, tumor wall disruption, and vascular invasion (Fig. 1), were collected.

Treatment decisions were made through consensus among attending physicians, considering patients’ performance status, organ functions, comorbidities, and the preferences of the patients and their families. Clinical information, including age, sex, vital signs, underlying liver disease, Child–Pugh score, hepatitis profile, date of treatment received (transarterial embolization or perihepatic packing), and dates of rHCC diagnosis and death, were collected.

For rHCC patients who underwent transarterial embolization (TAE), the intervention radiologists reviewed the location of treated vessels and angiographic images during the procedure.

### Transarterial embolization (TAE)

All TAE procedures were done via femoral artery puncture under local anesthesia. The location of the rHCC was identified through pre-procedure imaging. Angiography was used to determine the tumor’s arterial feeder. The decision of whether to perform lobar, segmental, or subsegmental embolization was made by the interventionists. In all cases, the embolic agent used was small gelfoam pledgets (1–3 mm) mixed with contrast media. The embolization procedure was considered successful if it resulted in bleeding cessation or the blockage of the tumor’s blood flow, as confirmed by post-embolization angiography images..

### Statistical analysis

Statistical analyses were performed using Stata 18 (StataCorp, College Station, Texas, USA). Frequency and percentage were used to describe categorical data, whereas mean and standard deviation or median and interquartile range (IQR) were used for numerical data, as appropriate. We examined the differences in all baseline characteristics, including imaging factors, across groups using either one-way ANOVA or the Chi-squared test. To diagnose the balance in baseline prognostic characteristics, we calculated the standardized difference (STD) for all groupwise comparisons, using it as an indicator of significant differences in pre-treatment prognostic factors (i.e., age, sex, hypovolemic shock, CTP score, and BCLC staging) [14]. An STD value greater than 0.1 or less than -0.1 was considered to indicate a significant difference.

Initially, we used Kaplan–Meier survival estimates to examine the crude survival distribution among the groups. To causally estimate comparative effectiveness, inverse probability treatment weighting (IPTW) was employed to balance differences in pre-treatment confounders across the treatment groups. Treatment weights were derived from a multinomial logistic regression that incorporated contextually-defined pre-treatment covariates: age, sex, hypovolemic shock, CTP score, and BCLC staging. Hypovolemic shock represents the result of tumor bleeding and indicates short-term survival for the patient [15]. Most HCC patients had underlying liver cirrhosis, which is generally evaluated with the CTP score. Hepatic failure is one of the important prognostic factors in HCC patients [13]. BCLC staging is one of the widely accepted guidelines for staging and treatment consideration for HCC. It considers patient performance status, liver function, and tumor burden. BCLC staging has been shown to correlate with the mortality of HCC patients [16]. IPTW was calculated from the inverse of the probability of receiving each specific treatment (e.g., the inverse of the probability of receiving TAE for patients who actually received TAE), which redistributes the original sample size across the treatment groups to create a balanced pseudopopulation. Weight trimming was carried out using Stürmer’s methods, specifically by omitting patients with a predicted treatment weight below the 5th percentile from each treatment group. Subsequently, we utilized multivariable flexible parametric survival regression with robust standard error to estimate the hazard ratio and 95% confidence intervals for all-cause mortality at 1 year. Covariate adjustment was performed for pre-treatment confounders that still exhibited an STD value above the threshold and other potential prognostic confounders (i.e., number of tumors, size of tumors, PV invasion, HV IVC invasion, location of bleeding, and presence of active contrast extravasation). Additionally, we calculated the restricted mean survival time (RMST) differences at 1 year for each comparison.

An exploratory analysis was conducted on the unweighted sample to identify significant imaging parameters that act as prognostic factors for 1-year survival. This was done by adjusting for treatment groups and other potential confounders using multivariable flexible parametric survival regression. Any factors identified would be incorporated as interactions with treatment modalities in the flexible parametric model of the weighted sample. This further examination would determine whether the selection of treatment modalities should be stratified accordingly.

## Results

**Fig. 2:**
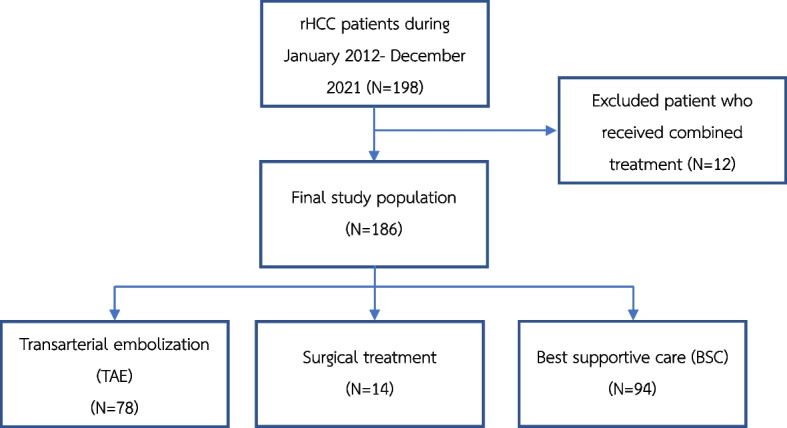
Study flow diagram of the patient cohort

Our study included 186 out of 198 rHCC patients at Maharaj Nakorn Chiang Mai Hospital from January 2012 to December 2021, excluding those who received combined treatment (surgery with TAE). The study flow diagram is presented in Fig. 2.

Among the 186 patients, 94 individuals (51%) received best supportive care. TAE was performed in 78 patients (42%) with a 100% success rate. Surgical management was administered to 14 patients (7%), including 4 wedge resections, 1 segmentectomy, 3 right hepatectomies, 2 left hepatectomies, and 4 perihepatic packings. None of the patients received combined systemic therapy such as TKI or PD-1/PD-L1 because, in our healthcare system, government insurance doesn’t cover these treatments for HCC, making them unaffordable for most patients.

Table 1 summarizes the demographic and clinical data of all 186 patients. The majority were men, with 151 out of 186 patients (81%). Liver function was classified as Child–Pugh class A for 9 patients (5%), class B for 97 patients (52%), and class C for 80 patients (43%). Sixty-two patients (33%) were in early or intermediate stages (BCLC A and B), while 124 patients (67%) were in advanced or terminal stages (BCLC C and D). The Child–Pugh score, BCLC staging, and total bilirubin were found to be significantly different among the three groups.

**Table 1:** Baseline clinical characteristics of the patient cohort classified by treatment groups

Characteristics	TAE (*n* = 78)		Surgery (*n* = 14)		Conservative (*n* = 94)		*P*-value
	*n*	(%)	*n*	(%)	*n*	(%)	
Age							
< 60 years	37	(47.4)	7	(50.0)	54	(57.5)	0.415
≥ 60 years	41	(52.6)	7	(50.0)	40	(42.5)	
Sex							
Female	16	(20.5)	4	(28.6)	15	(16.0)	0.467
Male	62	(79.5)	10	(71.4)	79	(84.0)	
CTP							
A	6	(7.7)	2	(14.3)	1	(1.0)	< 0.001
B	50	(64.1)	10	(71.4)	37	(39.4)	
C	22	(28.2)	2	(14.3)	56	(59.6)	
BCLC staging							
A or B	38	(48.7)	9	(64.3)	15	(16.0)	< 0.001
C or D	40	(51.3)	5	(35.7)	79	(84.0)	
Shock							
No shock	37	(47.4)	7	(50.0)	35	(37.2)	0.338
Shock	41	(52.6)	7	(50.0)	59	(62.8)	
Platelet							
< 75000	10	(12.8)	2	(14.3)	6	(6.4)	0.303
≥ 75,000	68	(87.2)	12	(85.7)	88	(93.6)	
Total bilirubin							
< 3	69	(88.5)	14	(100)	50	(53.2)	< 0.001
≥ 3	9	(11.5)	0	(0)	44	(46.8)	
Serum albumin							
> 2.8	33	(42.3)	10	(71.4)	40	(42.5)	0.111
≤ 2.8	45	(57.7)	4	(28.6)	54	(57.5)	

*CTP* Child-Turcotte-Pugh score, *TAE* Transarterial embolization, *BCLC* Barcelona clinic liver cancer

Table 2 summarizes the imaging characteristics of the study cohort. Of the 186 patients, 55 individuals (30%) had a single HCC, while 131 patients (70%) had multiple HCC. Tumor size was less than 5 cm in 27 out of 186 patients (15%). Portal vein invasion was found in 118 patients (63%), and hepatic vein invasion was found in 125 patients (67%). The right hepatic lobe was the most frequent location of the bleeding tumor, found in 116 patients (62%). The most common ancillary finding of rHCC in our cohort was a subcapsular location, observed in 184 patients (99%). A sentinel blood clot was present in 162 patients (87%), active contrast extravasation in 109 patients (59%), and focal wall disruption in 120 patients (65%). Table 2 summarizes the imaging characteristics of the study cohort, highlighting statistically significant differences in the number of tumors, portal vein invasion, and hepatic vein invasion among the three groups.

**Table 2:** Imaging characteristics of the patient cohort classified by treatment groups

Characteristics	TAE (*n* = 78)		Surgery (*n* = 14)		Conservative (*n* = 94)		*P*-value
	*n*	(%)	*n*	(%)	*n*	(%)	
Number of tumors							
Single	27	(34.6)	10	(71.4)	18	(19.2)	< 0.001
Multiple	51	(65.4)	4	(28.6)	76	(80.9)	
Size of tumors							
< 5 cm	11	(14.1)	3	(21.4)	13	(13.8)	0.746
≥ 5 cm	67	(85.9)	11	(78.6)	81	(86.2)	
PV invasion							
No	63	(80.8)	12	(85.7)	43	(45.7)	< 0.001
Yes	15	(19.2)	2	(14.3)	51	(54.3)	
HV or IVC invasion							
No	60	(76.9)	11	(78.6)	54	(57.5)	0.016
Yes	18	(23.1)	3	(21.4)	40	(42.5)	
Location of bleeding							
Left lobe	31	(39.8)	7	(50.0)	21	(22.4)	0.028
Right lobe	43	(55.1)	7	(50.0)	66	(70.2)	
Caudate lobe	4	(5.1)	0	(0)	2	(2.1)	
Both lobes	0	(0)	0	(0)	5	(5.3)	
Active contrast extravasation							
Absence	44	(56.4)	8	(57.1)	69	(73.4)	0.054
Presence	34	(43.6)	6	(42.9)	25	(26.6)	
Subcapsular							
Absence	0	(0)	0	(0)	2	(2.1)	0.372
Presence	78	(100)	14	(100)	92	(97.9)	
Wall disruption							
Absence	27	(34.6)	6	(42.9)	33	(35.1)	0.834
Presence	51	(65.4)	8	(57.1)	61	(64.9)	

*TAE* Transarterial embolization, *PV* Portal vein, *HV* Hepatic vein, *IVC* Inferior vena cava

**Fig. 3:**
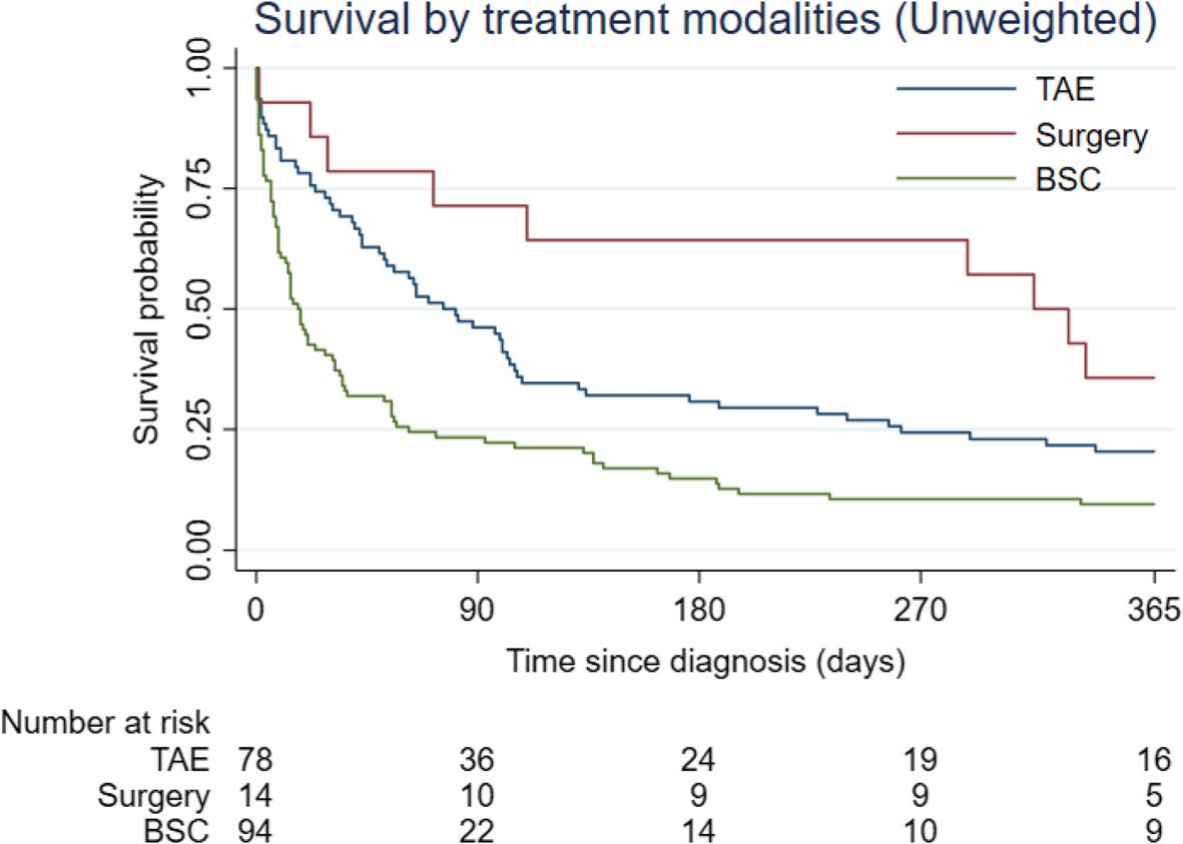
Kaplan–Meier curves classified by types of treatment received in the original cohort. Abbreviations**:** BSC, best supportive care; TAE, transarterial embolization

There were 12 patients in the TAE group and only one patient in the surgery group, who died during the same hospitalization as their treatment. Fifty-nine patients in the BSC group died from hypovolemic shock in the hospital. The exact cause of death could not be defined for the remaining patients in both the BSC group and other groups. The Kaplan–Meier curves demonstrated 90-day and 1-year mortality rates for all rHCC patients at 64% and 84%, respectively. When comparing the three groups, the surgery group had the lowest 1-year mortality rate, followed by the TAE group and the BSC group (64%, 80%, and 91%, respectively) (Fig. 3).

**Fig. 4:**
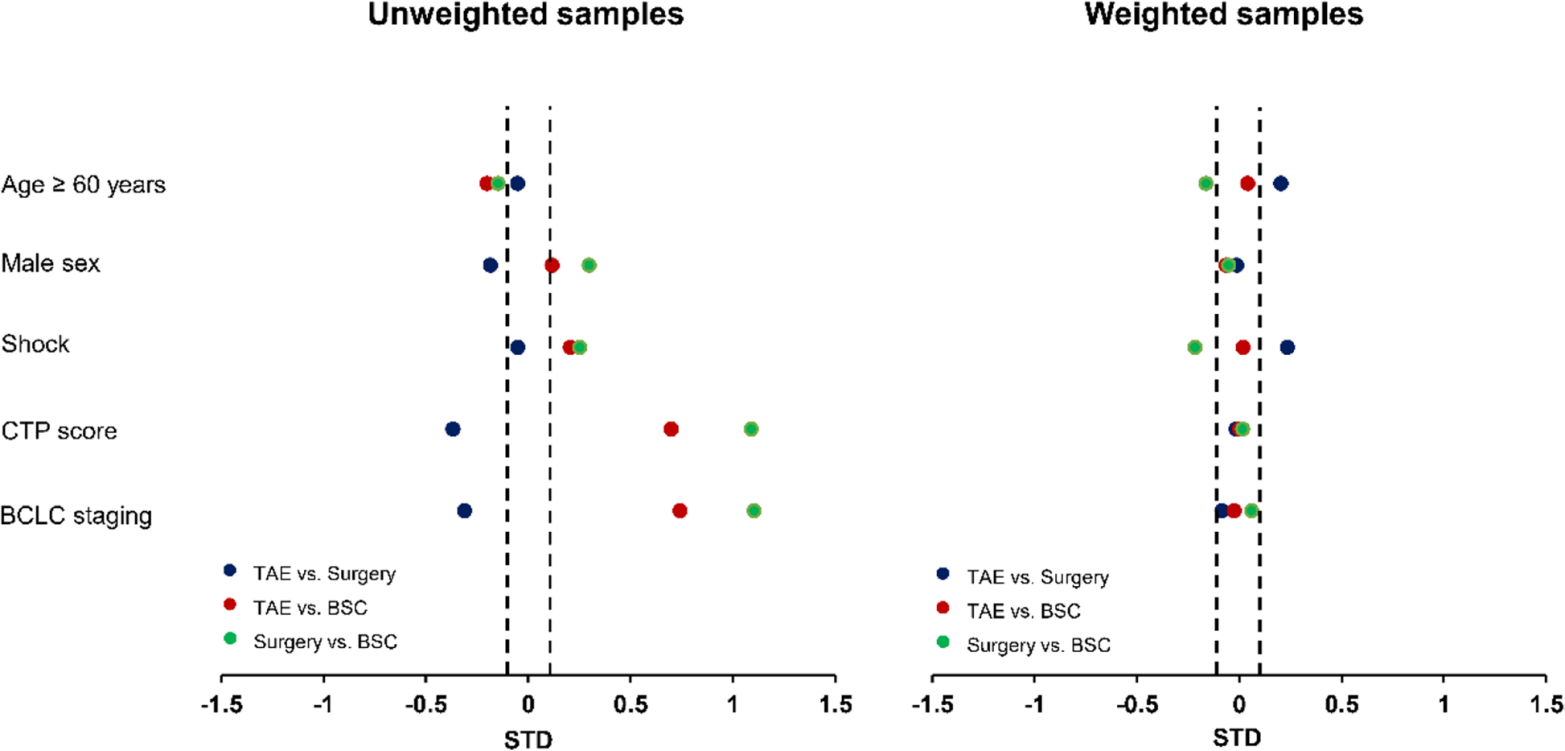
Differences in pre-treatment prognostic characteristics for each pairwise comparison in the unweighted and the weighted samples. Abbreviations: BSC, best supportive care; BCLC, Barcelona clinic liver cancer staging; CTP, Child-Turcotte-Pugh; STD, standardized difference; TAE, transarterial embolization

According to the pre-treatment prognostic factors shown in Table 1 and Fig. 4, the BSC group exhibited significantly higher CTP scores and more advanced BCLC staging compared to the TAE and surgery groups. Meanwhile, the TAE group showed significantly higher CTP scores and more advanced BCLC staging compared to the surgery group. These findings confirm the baseline imbalance of pre-treatment covariates across groups. Five patients in the TAE group and two patients in the conservative group showed extreme weights and were excluded from the analysis. In the weighted pseudopopulation, the final approximate number of patients within each treatment group was as follows: 184 in the TAE group, 171 in the surgery group, and 186 in the BSC group. After IPTW, pre-treatment factors became more balanced among the groups, especially CTP and BCLC staging. However, some characteristics remained different, such as age ≥ 60 years and shock (Fig. 4 and Supplementary Table S1). There was also a residual difference in the remaining imaging characteristics that were not included in the treatment weights model (Supplementary Table S1). All of these factors, along with age and shock, were subsequently adjusted in the weighted analysis model.

**Fig. 5:**
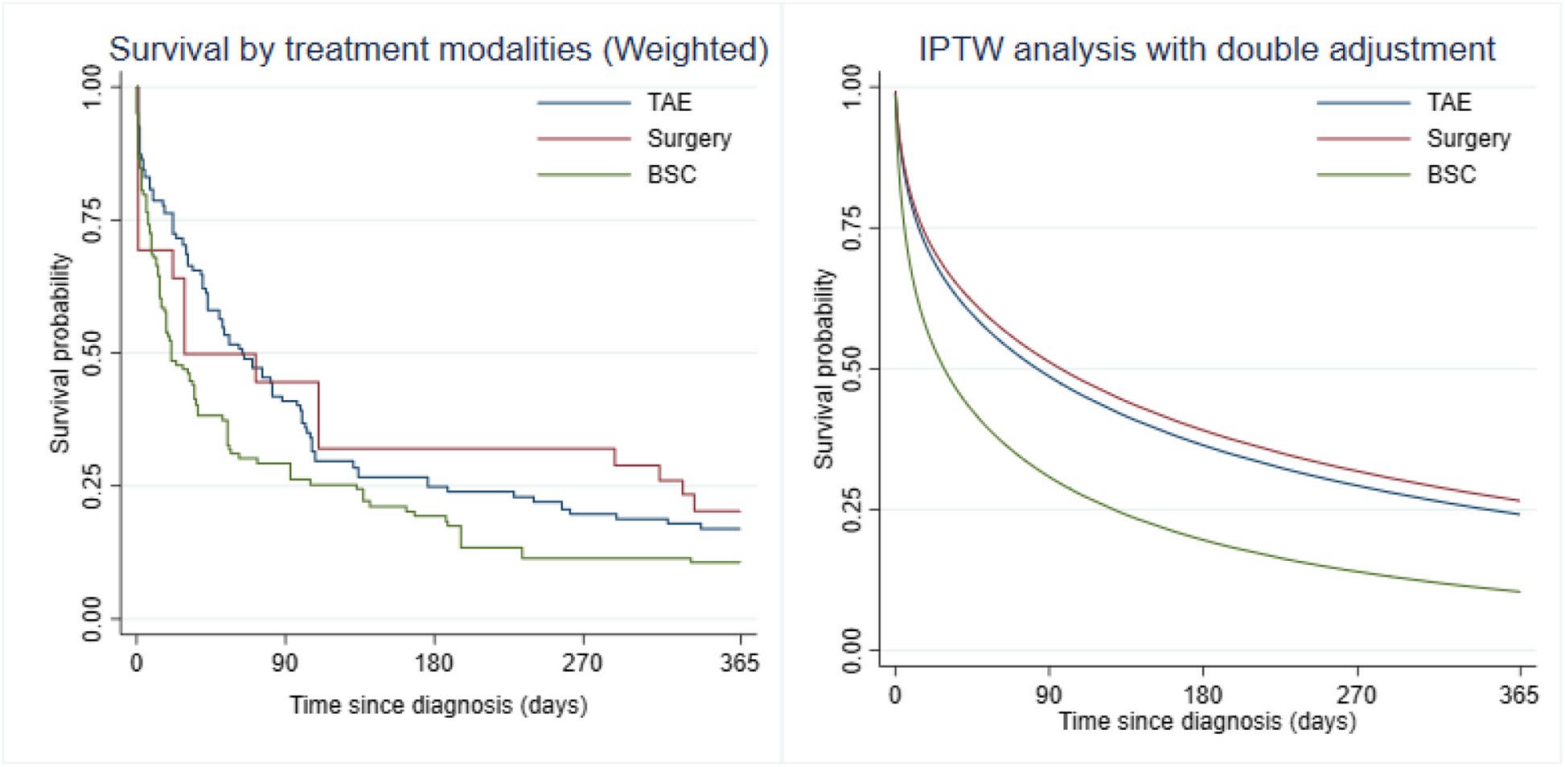
Kaplan–Meier curves classified by types of treatment received in the original cohort and the doubly-robust adjusted survival curves in the weighted samples. Abbreviations: BSC, best supportive care; TAE, transarterial embolization

**Fig. 6:**
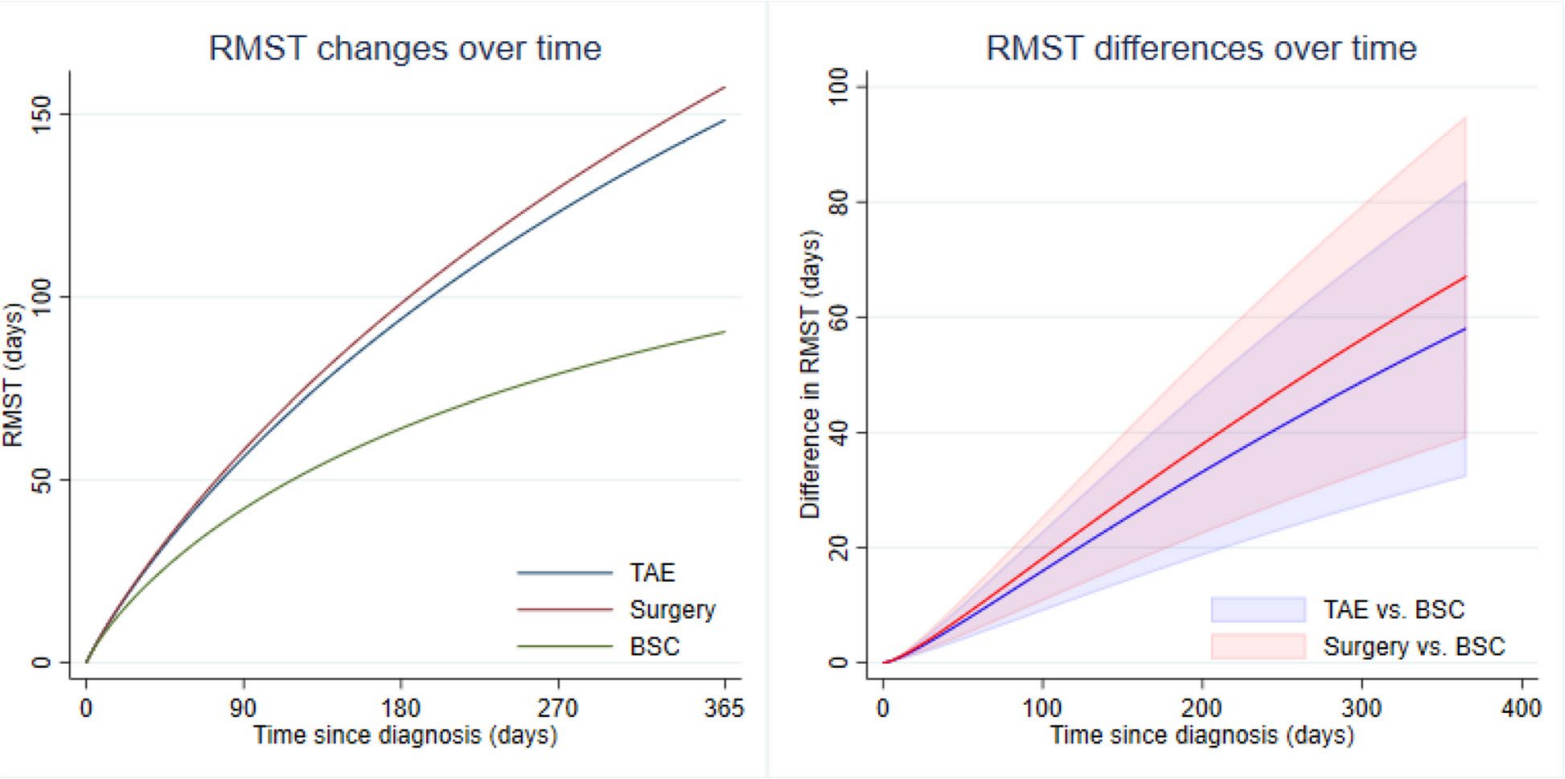
Changes in the restricted mean survival time (RMST) and restricted mean survival time differences over time estimated from the flexible parametric model in the weighted samples. Abbreviations: BSC, best supportive care; RMST, restricted mean survival time; TAE, transarterial embolization

After IPTW, the surgery and TAE groups showed a significant decrease in 1-year mortality compared with the BSC group. However, there was no statistically significant difference in 1-year mortality between the surgery and TAE groups (Fig. 5). Similar results were obtained using weighted flexible parametric survival regression adjusted by confounding variables (Fig. 5). When compared to BSC, both the TAE and surgery groups showed significantly lower 1-year mortality rates (TAE HR 0.56, 95% CI 0.33–0.96; surgery HR 0.52, 95% CI 0.28–0.95). Additionally, both the TAE and surgery groups exhibited, on average, two months longer life expectancy during the first year after the initial treatment compared to BSC (TAE + 55.40, 95% CI 30.18–80.63 days; surgery + 68.43, 95% CI 38.77–98.09 days) (Table 3 and Fig. 6).

**Table 3:** Comparative effectiveness among different treatment modalities for ruptured HCC

1-year mortality	Adjusted estimates^a^	95% CI	*P*-value
Hazard ratio (HR)			
TAE	0.56	0.33, 0.96	0.033
Surgery	0.52	0.28, 0.95	0.034
BSC	1.00	Reference	
RMST difference			
TAE	+ 55.40	30.18, 80.63	< 0.001
Surgery	+ 68.43	38.77, 98.09	< 0.001
BSC	0.00	Reference	

*HR* hazards ratio, *RMST* restricted mean survival time, *TAE* Transarterial embolization, *BSC* Best supportive care, ^a^Adjusted by potential confounders: age, shock, number of tumor, tumor size, portal vein invasion, hepatic vein or IVC invasion, platelet, location of bleeding, and active extravasation of contrast

After an exploratory analysis of imaging parameters as prognostic factors for rHCC patients, the analysis, adjusted for treatment modalities and potential confounders, revealed that the presence of active contrast extravasation (HR 1.57, 95% CI 1.04–2.35) and bleeding of the tumor in both hepatic lobes (HR 2.97, 95% CI 1.08–8.19) were independent poor prognostic factors for survival at 1 year. Other imaging factors, such as vascular invasion, subcapsular location, tumor wall disruption, number, and size of the tumor, were not statistically significant (Table 4).

**Table 4:** Exploratory analysis of imaging parameters as prognostic factors for survival at 1 year adjusted for treatment modalities and potential prognostic factors using unweighted samples

1-year mortality	Univariable analysis			Multivariable analysis		
	uHR	95% CI	*P*-value	aHR	95% CI	*P*-value
Number of tumors						
Single	1.00	Ref		1.00	Ref	
Multiple	1.47	1.02, 2.13	0.038	1.17	0.77, 1.78	0.453
Size of tumors						
< 5 cm	1.00	Ref		1.00	Ref	
≥ 5 cm	0.99	0.63, 1.55	0.954	1.05	0.63, 1.76	0.842
PV invasion						
No	1.00	Ref		1.00	Ref	
Yes	1.50	1.09, 2.07	0.017	0.93	0.61, 1.44	0.758
HV or IVC invasion						
No	1.00	Ref		1.00	Ref	
Yes	1.51	1.09, 2.10	0.014	0.98	0.64, 1.48	0.917
Location of bleeding						
Left lobe	1.00	Ref		1.00	Ref	
Right lobe	1.30	0.92, 1.83	0.137	1.28	0.88,1.87	0.195
Caudate lobe	0.67	0.24, 1.85	0.437	0.61	0.21,1.82	0.377
Both lobes	2.53	1.00, 6.42	0.051	2.97	1.08,8.19	0.035
Active contrast extravasation						
Absence	1.00	Ref		1.00	Ref	
Presence	1.30	0.93, 1.80	0.121	1.57	1.04,2.35	0.030
Subcapsular location						
Absence	1.00	Ref		1.00	Ref	
Presence	0.68	0.17, 276	0.594	0.90	0.20,4.07	0.893
Wall disruption						
Absence	1.00	Ref		1.00	Ref	
Presence	1.05	0.76, 1.47	0.763	0.95	0.64,1.41	0.793

*CI* confidence interval, *aHR* adjusted hazard ratio, *uHR* unadjusted hazard ratio, *TAE* Transarterial embolization, *PV* Portal vein, *HV* Hepatic vein, *IVC* Inferior vena cava

Adjusted by treatment modalities, age, sex, shock, CTP, and BCLC staging

**Fig. 7:**
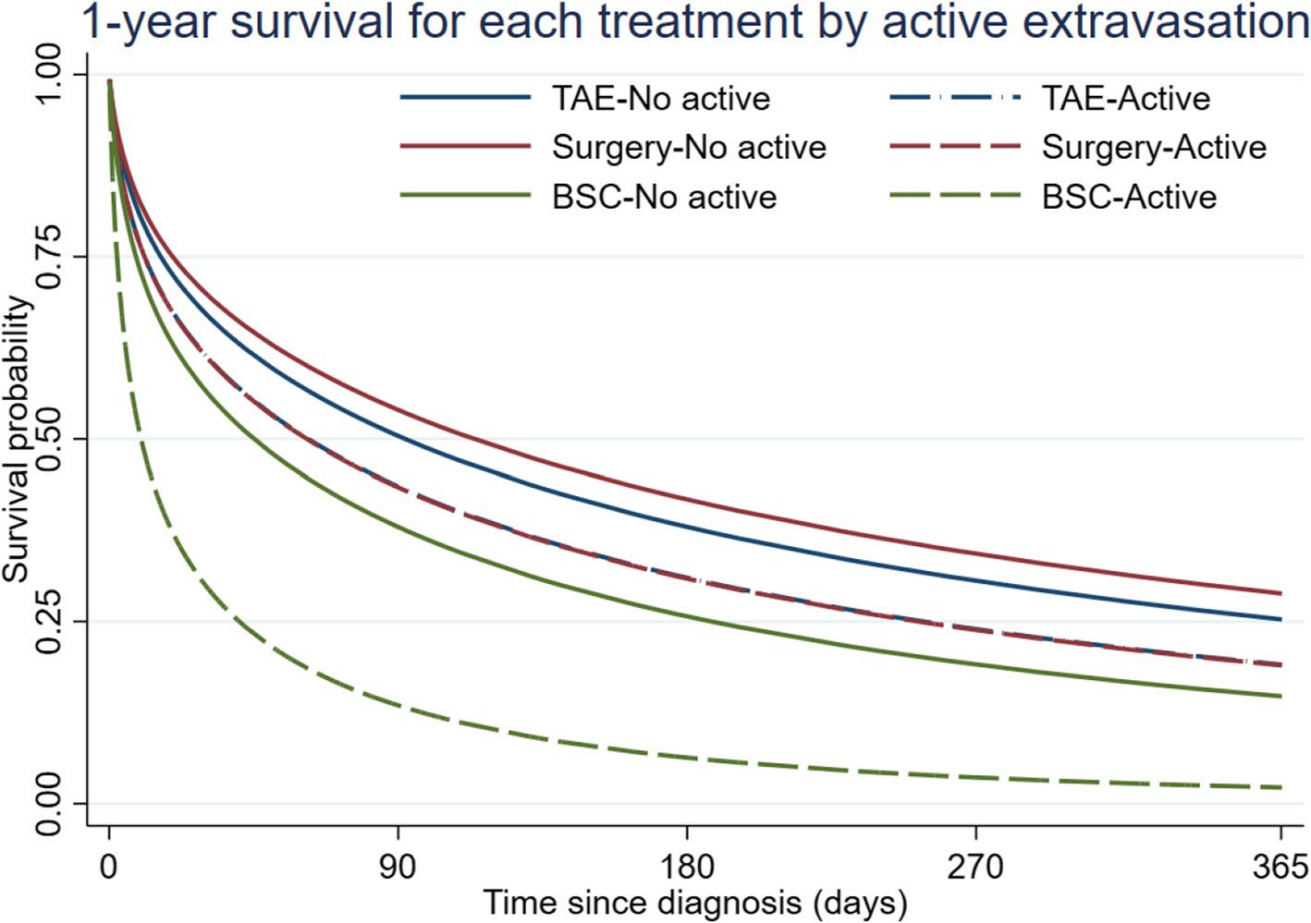
Doubly-robust adjusted survival curves in the weighted sample by treatment received stratified by active contrast extravasation. Abbreviations: BSC, best supportive care; TAE, transarterial embolization

The factor-treatment interaction analysis was feasible only for the presence of active contrast extravasation. The treatment-specific survival curves demonstrated that both TAE and surgery significantly improved the 1-year survival rate compared to BSC, regardless of the presence of active contrast extravasation (Fig. 7). For TAE and surgery, no significant interaction with the presence of active contrast extravasation was observed, while a significant difference in the 1-year survival was noted between patients who received BSC with active contrast extravasation and those without it (*P* < 0.001).

## Discussion

### Survival rate of rHCC patients and treatment benefits

Ruptured hepatocellular carcinoma (rHCC) is a fatal complication of HCC. Our cohort exhibited a 90-day mortality rate of 64% and a 1-year mortality rate of 84%. These findings align with previous reports indicating in-hospital mortality rates for rHCC ranging from 32 to 75% [11, 17–19]. When comparing the three treatment options, the surgery group demonstrated a significantly higher 1-year survival rate (36%) compared to TAE (20%) and BSC (9%), as illustrated in Fig. 3. Our results are consistent with those of Hsueh et al., who reported a 1-year survival rate of 28% in the TAE group and 62.2% in the surgery group [20]. We believe that these results may be attributed to baseline imbalances in liver function and tumor burden among the three groups, influencing the treatment decisions of attending physicians.

In our study, we included patient age, sex, hypovolemic shock, liver function (Child–Pugh score), tumor stage (BCLC), tumor size, vascular invasion, and the location of the bleeding tumor as pre-treatment covariates. These factors were adjusted using IPTW. In comparison to BSC, both the TAE and surgery groups demonstrated higher survival probabilities. Furthermore, the restricted mean survival time in both the TAE and surgery groups exceeded that in the BSC group. Our study confirmed the benefits of TAE and surgery in rHCC patients, extending survival time by approximately 2 months compared to best supportive care. Since surgery is a riskier procedure compared to TAE, it is typically considered for patients with good overall health, characterized by good liver function and a low tumor burden [21, 22]. After adjusting for these pretreatment confounding factors, we demonstrated that the benefits of both treatment modalities appear to be similar.

Consistent with previous reports, our study reaffirms the poor prognosis associated with best supportive care in rHCC [8, 20, 23]. Currently, best supportive care is the only viable option for patients in a moribund state, with poor liver function and locally advanced tumors ineligible for transcatheter embolization or surgery[3, 24, 25]. TAE is a minimally invasive procedure effective in controlling acute tumor bleeding by blocking the hepatic arterial blood supply. It requires only local anesthesia with femoral artery puncture, resulting in a low complication rate [26, 27]. On the other hand, emergency liver resection may offer the potential for a cure for rHCC [19, 28]. Numerous studies consistently show that surgery provides a better long-term survival rate compared to TAE [3, 11, 29, 30]. In our study, after adjusting for pretreatment confounding factors, we found that TAE and surgery demonstrated similar 1-year survival rates in patients with rHCC (Fig. 5). In the emergency setting of rHCC, we recommend considering TAE as the initial intervention due to its less invasive nature. If the tumor is suitable for surgical intervention, a staged hepatectomy following TAE should be pursued to attain long-term survival outcomes.

*Imaging predicting factor of rHCC patientsThe multivariable analysis* revealed that a bleeding tumor in both hepatic lobes (HR 2.97) and the presence of active contrast extravasation (HR 1.57) are statistically significant poor prognostic factors. Bleeding tumors in both lobes are rare but fatal. In our study, one case was presented with DVT and received anticoagulation treatment. Subsequently, the patient developed abdominal pain. A CT scan revealed multiple HCCs in both lobes of the liver, accompanied by hemoperitoneum with active extravasation from several tumors in both lobes. Bleeding of a tumor in both hepatic lobes can lead to significant blood loss, making bleeding control more challenging and increasing the risk of liver insufficiency following the treatment procedure [31]. Additionally, active contrast extravasation directly indicates ongoing tumor bleeding [32]. These two imaging findings likely indicate a more severe level of bleeding from HCC. The treatment-specific survival curve in the subgroup of patients with active contrast extravasation revealed that both TAE and surgery can significantly improve the 1-year survival rate in this poor prognosis group of patients. This encourages more active management for rHCC patients with active contrast extravasation.

### Strength and limitations

The strength of this study lies in our comprehensive comparison of the three treatment modalities for rHCC patients, prioritizing the minimization of pre-treatment covariates’ effects. We effectively demonstrated the advantages of surgical and TAE management for rHCC patients, particularly in terms of time, which is crucial for late-stage cancer patients.

Our study also had some limitations. Firstly, its retrospective design may be subject to inherent biases and limitations associated with retrospective analyses. Additionally, there is a lack of data on the exact cause of death, adverse events, and risk of liver failure in many cases. This absence is due to patients and their families preferring end-of-life care at home, making it difficult to verify these adverse events and the exact cause of death. Secondly, relying solely on survival time to justify treatment effectiveness may not be holistic. Other relevant endpoints that also affect patients should be considered. Post-treatment adverse events (e.g., incidence of liver failure) and quality of life data were not available for most cases. This study is also limited by insufficient data regarding fluid resuscitation and blood transfusion, which may serve as confounding factors and affect treatment outcomes. However, we believe that transfusion treatment, as a standard of care, should exhibit minimal variation among patients.

## Conclusions

TAE and surgical treatments provide comparable survival benefits for patients with rHCC. These interventions extend survival time by approximately 2 months compared to best supportive care. We strongly recommend active management for all rHCC patients whenever possible.

### Supplementary Information

Supplementary Material 1.

Supplementary Material 2.

Supplementary Material 3.

## Data Availability

The dataset generated and analyzed during current study are included in the supplementary files.

## Data availability

Dataset in this paper has been uploaded within the “supplementary files”
