# Supplementary material for: Comparative analysis among therapeutic modalities in ruptured hepatocellular carcinoma and identification of imaging predictors for survival
Source: BMC Cancer. 2024 Aug 26;24:1045. doi: 10.1186/s12885-024-12829-y (PMC11346290; doi:10.1186/s12885-024-12829-y)
Supplement: Supplementary file 1 — Supplementary Material 1. [file 12885_2024_12829_MOESM1_ESM.docx]

**Supplementary Table S1** Baseline clinical characteristics of the patient cohort were classified by treatment groups before and after applying inverse probability of treatment weights (IPTWs).

| Characteristics | Unweighted (original) samples | | | | | | Weighted samples | | |
| --- | --- | --- | --- | --- | --- | --- | --- | --- | --- |
|  | TAE  (n=78) | | Surgery  (n=14) | | Conservative (n=94) | | TAE  (n=184) | Surgery  (n=171) | Conservative (n=186) |
|  | n | (%) | n | (%) | n | (%) | (%) | (%) | (%) |
| Age |  |  |  |  |  |  |  |  |  |
| < 60 years | 37 | (47.4) | 7 | (50.0) | 54 | (57.5) | (53.8) | (43.5) | (51.7) |
| ≥ 60 years | 41 | (52.6) | 7 | (50.0) | 40 | (42.5) | (46.2) | (56.5) | (48.3) |
| Sex |  |  |  |  |  |  |  |  |  |
| Female | 16 | (20.5) | 4 | (28.6) | 15 | (16.0) | (19.2) | (19.7) | (21.8) |
| Male | 62 | (79.5) | 10 | (71.4) | 79 | (84.0) | (80.8) | (80.3) | (78.2) |
| CTP |  |  |  |  |  |  |  |  |  |
| A | 6 | (7.7) | 2 | (14.3) | 1 | (1.0) | (3.3) | (6.2) | (3.0) |
| B | 50 | (64.1) | 10 | (71.4) | 37 | (39.4) | (53.9) | (48.8) | (54.4) |
| C | 22 | (28.2) | 2 | (14.3) | 56 | (59.6) | (42.8) | (44.9) | (42.6) |
| BCLC staging |  |  |  |  |  |  |  |  |  |
| A or B | 38 | (48.7) | 9 | (64.3) | 15 | (16.0) | (32.1) | (36.3) | (33.3) |
| C or D | 40 | (51.3) | 5 | (35.7) | 79 | (84.0) | (67.9) | (63.7) | (66.7) |
| Shock |  |  |  |  |  |  |  |  |  |
| No shock | 37 | (47.4) | 7 | (50.0) | 35 | (37.2) | (42.7) | (31.1) | (41.7) |
| Shock | 41 | (52.6) | 7 | (50.0) | 59 | (62.8) | (57.3) | (68.9) | (58.3) |
| Platelet |  |  |  |  |  |  |  |  |  |
| <75000 | 10 | (12.8) | 2 | (14.3) | 6 | (6.4) | (13.1) | (44.9) | (7.7) |
| ≥75000 | 68 | (87.2) | 12 | (85.7) | 88 | (93.6) | (86.9) | (55.1) | (92.3) |
| Total bilirubin |  |  |  |  |  |  |  |  |  |
| <3 | 69 | (88.5) | 14 | (100) | 50 | (53.2) | (83.0) | (100) | (64.2) |
| ≥3 | 9 | (11.5) | 0 | (0) | 44 | (46.8) | (17.0) | (0) | (35.8) |
| Serum albumin |  |  |  |  |  |  |  |  |  |
| >2.8 | 33 | (42.3) | 10 | (71.4) | 40 | (42.5) | (32.7) | (47.0) | (51.1) |
| ≤2.8 | 45 | (57.7) | 4 | (28.6) | 54 | (57.5) | (67.3) | (53.0) | (48.9) |
| Number of tumors | | | | | | | | | |
| Single | 27 | (34.6) | 10 | (71.4) | 18 | (19.2) | (31.6) | (63.2) | (19.9) |
| Multiple | 51 | (65.4) | 4 | (28.6) | 76 | (80.9) | (68.4) | (36.8) | (80.1) |
| Size of tumors |  |  |  |  |  |  |  |  |  |
| <5 cm | 11 | (14.1) | 3 | (21.4) | 13 | (13.8) | (15.4) | (15.5) | (14.8) |
| ≥5 cm | 67 | (85.9) | 11 | (78.6) | 81 | (86.2) | (84.6) | (84.5) | (85.2) |
| PV invasion |  |  |  |  |  |  |  |  |  |
| No | 63 | (80.8) | 12 | (85.7) | 43 | (45.7) | (77.5) | (84.3) | (55.4) |
| Yes | 15 | (19.2) | 2 | (14.3) | 51 | (54.3) | (22.5) | (15.7) | (44.6) |
| HV IVC invasion |  |  |  |  |  |  |  |  |  |
| No | 60 | (76.9) | 11 | (78.6) | 54 | (57.5) | (71.6) | (81.2) | (64.3) |
| Yes | 18 | (23.1) | 3 | (21.4) | 40 | (42.5) | (28.4) | (18.8) | (35.7) |
| Location of bleeding | | | | | | | | | |
| Left lobe | 31 | (39.8) | 7 | (50.0) | 21 | (22.4) | (45.9) | (62.3) | (25.6) |
| Right lobe | 43 | (55.1) | 7 | (50.0) | 66 | (70.2) | (47.4) | (37.7) | (65.9) |
| Caudate lobe | 4 | (5.1) | 0 | (0) | 2 | (2.1) | (6.7) | (0) | (1.5) |
| Both lobes | 0 | (0) | 0 | (0) | 5 | (5.3) | (0) | (0) | (7.0) |
| Active contrast extravasation | | | | | | | | | |
| Absence | 44 | (56.4) | 8 | (57.1) | 69 | (73.4) | (53.9) | (29.2) | (77.3) |
| Presence | 34 | (43.6) | 6 | (42.9) | 25 | (26.6) | (46.1) | (70.8) | (22.7) |
| Subcapsular |  |  |  |  |  |  |  |  |  |
| Absence | 0 | (0) | 0 | (0) | 2 | (2.1) | (0) | (0) | (1.8) |
| Presence | 78 | (100) | 14 | (100) | 92 | (97.9) | (100) | (100) | (98.3) |
| Wall disruption |  |  |  |  |  |  |  |  |  |
| Absence | 27 | (34.6) | 6 | (42.9) | 33 | (35.1) | (32.1) | (55.9) | (41.7) |
| Presence | 51 | (65.4) | 8 | (57.1) | 61 | (64.9) | (67.9) | (44.1) | (58.3) |

Characteristics included in the pre-treatment weighting model were highlighted in light yellow. Imaging characteristics not included in the pre-treatment weighting model were highlighted in light green. Platelet, total bilirubin, and serum albumin were not included in the analysis due to their collinearity with the Child-Turcotte-Pugh score. **Abbreviations:** BCLC, Barcelona clinic liver cancer; CTP, Child-Turcotte-Pugh score; HV, Hepatic vein; IVC, Inferior vena cava; PV, Portal vein; TAE, Transarterial embolization.
