# Supplementary material for: Comparative analysis among therapeutic modalities in ruptured hepatocellular carcinoma and identification of imaging predictors for survival
Source: BMC Cancer. 2024 Aug 26;24:1045. doi: 10.1186/s12885-024-12829-y (PMC11346290; doi:10.1186/s12885-024-12829-y)
Supplement: Supplementary file 2 — Supplementary Material 2. [file 12885_2024_12829_MOESM2_ESM.docx]

**Supplementary Table 1** Differences in pre-treatment prognostic characteristics for each pairwise comparison in the unweighted and the weighted samples

|  | Unweighted | | | Weighted | | |
| --- | --- | --- | --- | --- | --- | --- |
|  | TAE vs Surgery | TAE vs BSC | Surgery vs BSC | TAE vs Surgery | TAE vs BSC | Surgery vs BSC |
| Age ≥60 years | -0.0501988 | -0.2002856 | -0.1465643 | 0.2038051 | 0.0426311 | -0.1616594 |
| Male sex | -0.183685 | 0.1174782 | 0.2992606 | -0.0120786 | -0.0649264 | -0.0517745 |
| Shock | -0.0501988 | 0.2063621 | 0.2539398 | 0.2352537 | 0.0194192 | -0.2160503 |
| CTP | -0.3658618 | 0.7008539 | 1.091642 | -0.0144924 | 0.002632 | 0.0170327 |
| BCLC staging | -0.3112517 | 0.7431831 | 1.104657 | -0.0851867 | -0.0248985 | 0.0607535 |
